# Methods for analysing citizens’ attitudes: a hypothetical Italian referendum about the membership of the European Union as a case study

**DOI:** 10.1007/s11135-021-01201-y

**Published:** 2021-07-13

**Authors:** Marino De Luca

**Affiliations:** Department of Politics - School of Law, Politics and Sociology of the University of Sussex, Falmer, Brighton, UK

**Keywords:** Euroscepticism, Italy, Referendum, EES survey, CATPCA

## Abstract

The European Union is an unprecedented unification project that successfully preserves political peace and integrates Europe’s countries into a supra-national model. However, recent economic and political crises have shown that there are institutional problems that have undermined the EU and lost the trust of many citizens. In Italy after the ‘political earthquake’ of the 2013 national elections, the party system suffered a further shock in 2018 with the consolidation of the centre-right and Five Star Movement as the main competing political actors. In this context, the relationship with the EU has undergone numerous tensions, impacting directly on Italian public opinion and its perception of European institutions. This paper investigates whether and how the ‘exit’ issue from the EU affects Italian citizens, particularly how they react to a UK-style hypothetical referendum on leaving the EU. By analysing a 2019 voter study, it tries to identify clusters of Italian citizens by their attitude to European policies and a possible EU referendum.

## Introduction

The European Union (EU) is currently encountering one of the most uncertain periods since its birth. Although the EU project has been very successful at maintaining political peace, recent crises have displayed the institution’s fragility.

The EU’s economic vision, border protection and institutional legitimacy have become the main issues in Eurosceptic political strategies and they have consequently left their mark on public opinion (De Vries 2018).

In this context, although the UK is an outlier when it comes to exit scepticism (Hobolt 2016) and there are different reasons why people would like to leave the EU in different countries (De Vries 2018; Ejrnæs and Jensen 2019, 2021), the UK’s 2016 vote to exit is the first real example shedding light on the consequences of the intensifying Eurosceptic sentiment (De Luca 2019). Since then, the fear of a likely ‘contagion’ in other countries (Walter 2020, 2021) has persistently spread to the point of worrying Rome seriously.

Although the early years were characterised by strong public support in Italy towards European integration (Conti 2006; Isernia 2008), by the early 1990s Italian political parties had developed relevant Eurosceptic positions (Quaglia 2008, 2011).

A general decline in EU trust has occurred in the last decade. Still, the negative peaks in EU support have occurred with two acute crises (Harteveld et al. 2013; Dellmuth and Tallberg 2015; Isani and Schlipphak 2017; Conti et al. 2020b). First, after the 2010–2011 economic crisis and the subsequent technocratic government, led by Mario Monti, the EU was ‘blamed’ by Italians for the austerity measures realised against member states (Conti et al. 2020a). Second, the 2015–2017 immigration crisis highlighted EU governance limitations in containing the enormous numbers of immigrant arrivals from Africa and the Middle East area. The asylum rules provided by the Dublin regulation quickly became, in Italian public perception, too expensive compared to the benefits of EU membership (Ambrosini 1995; Dixon et al. 2018; Geddes and Pettrachin 2020).

Between these two crises, there was the ‘political earthquake’ of the 2013 national elections (Chiaramonte and Sio 2013); with the rise in the anti-establishment Five Star Movement (M5S), the Italian party system changed from the bipolarity of the Second Republic into a tripolar system (Ceccarini and Bordignon 2016; Conti and Memoli 2015; Salvati 2019).

A further violent shock in 2018 consolidated the M5S and the centre-right League as the main competing political actors. In this political context, M5S and the League struck an agreement forming the first coalition government which excluded the ‘mainstream’ parties, allowing them to mix several policies historically connected with both the right wing and the left. In just a few weeks during summer 2019, under Salvini’s leadership, the League revoked its support of the cabinet, provoking a government crisis that resulted in the formation of a new cabinet between M5S and the Democratic Party (PD), led again by the PM Giuseppe Conte (Gattinara and Froio 2019; Salvati 2020).

In this new scenario, the position of the League became more critical for the EU in relation to pushing Italian public opinion to have more regard for national affairs. Moreover, new political forces have EU-exit agendas, such as ‘Italexit’, the new senator Gianluigi Paragone’s party. The economy, immigration and Covid-19 crises have proven to be real pressure tests for the Brussels–Rome relationship. Trust in the EU seems to have deteriorated, raising concerns about possible severe consequences for the stability of EU countries. The most crucial factor that has been brought into play is that Eurosceptic positions now have a stronger voice than the pro-EU forces.

For this reason, the Italian public’s opinion towards this relationship is becoming more and more like a litmus test of the national elite’s consensus and its policy competition.

In order to understand these changes, the research question to pay close attention to is ‘How will Italian citizens react in the face of a hypothetical referendum on exiting the EU modelled on the UK referendum?’.

Although from a legal viewpoint the possibility of leaving the EU raises several constitutional queries that the Brexit model does not solve—in particular, the prospect of a legal ‘Italexit’ no longer appears to be unimaginable (Miglio 2019). Moreover, even if at the moment ‘Italexit’ is not on the public opinion agenda, several scholars argue that today Italexit is not a completely theoretical possibility (Baccaro et al. 2020).^1^ For this reason, this paper’s aim is to evaluate the relationship between Eurosceptic attitudes in Italy and political phenomena such as a referendum on Italexit after the precipitation of the situation in the UK. Italexit is a term used to describe the possible exit of Europe’s third-largest economy from the EU (Fingleton 2020) as a solution to Italy’s economic and social issues, particularly related to immigration (Simionescu 2021).

To understand this topic, this paper analyses the European Election Studies (EES) Voter Study 2019 in addition to trying to identify clusters of Italian citizens according to their attitudes to European and national issues and a referendum with the option of leaving the EU.

This paper is divided into five sections. The first section analyses Euroscepticism in relation to public opinion. The second section presents the data and the method used to discuss the variable selection used in the categorical principal component analysis (CATPCA). In the third section, the results of CATPCA are considered using the components involved in a cluster analysis of Italian citizens. The fourth section aims to understand the Italian approach to a referendum on exiting the EU. Finally, the fifth section comprises the conclusion.

## Mass level Euroscepticism

A number of important recent surveys centred on the EU and essential attitudes towards EU institutions (i.e. Eurobarometer, EES, etc.) have enabled us to better understand public Euroscepticism. The factors that seem to power it include emotional reactions to national politics and institutions, fears about how an integration process may affect national identity, a dislike of supra-national institutions and utilitarianism based on personal interest (Hobolt and De Vries 2016).

Studies in the 1990s, in which individual cost-benefits encouraged explanations that depend on strong links between support for EU integration and greater affluence, focused on a utilitarian interpretation (Anderson and Reichert 1995; Gabel 1998). The EU was generally not seen as a major risk to each country’s national and cultural identity, underlining how the economic costs and benefits were vital in forming public opinion towards the European integration process (McLaren 2002, 2007). Europeans who believed in a strong economic future saw European integration positively and those who were more fearful were also more Eurosceptic. Another key element in EU support is how educated citizens are, as recent studies have found (Ejrnæs and Jensen 2021; Hakhverdian et al. 2013). Recent Eurozone studies demonstrate that where the economic cost–benefit is meaningful, there is a firmer utility aspect in public opinion (Hobolt and Wratil 2015). Others describe how economic conditions during the 2007–2010 global economic crisis and the ‘austerity’ measures that resulted drove citizens’ attitudes towards the EU institutions (Gomez 2015). Economic factors such as unemployment and a higher interest rate therefore suggest a high correlation with weaker levels of EU support. Furthermore, Europeans are now more responsive to economic issues as a result of the crisis, although this seems to be less visible in countries with intensive social policies than in those with fewer welfare protections (Anderson and Hecht 2014).

Although the determinants often complement each other (Garry and Tilley 2009; Hooghe 2007; Kuhn et al. 2014; McLaren 2007), national identity and political institutions have been key in accounting for public Euroscepticism (Serricchio et al. 2013). The idea of identity had already been defined a number of times as acting in contrast to economic factors (McLaren 2006). The supra-national model seems to dissolve national identity which leads citizens with a solid national identity to look at EU integration less positively (Carey 2002; Hooghe and Marks 2004).

Support for national identity factors seemed to shift with a second pivotal moment. The European refugee crisis that peaked in 2015–2017 created a high level of tension on Europe’s borders upon displaying the inability of the EU to manage the emergency. Non-economic factors, such as cultural/national identity and security, that immigration seemed to threaten, were directly linked to this situation. These are factors that, as we have seen, are likely to affect citizens’ attitude towards EU institutions (McLaren 2002). More widely, those who hold Eurosceptic views tend to show resentment towards immigrants (Vreese and Boomgaarden 2006). In addition, a narrow idea of a national/local identity is more likely to encourage a Eurosceptic position (Hooghe and Marks 2004).

Moreover, in southern member states, the 2015–2017 refugee crisis did increase citizens’ distrust in the benefits of EU membership. In Italian public opinion, the Dublin regulation’s asylum rules quickly became regarded as too expensive when weighed up against what the EU membership offered (Dixon et al. 2018; Geddes and Pettrachin 2020).

However, public opinion also seems easily swayed by the national political context (Hobolt and De Vries 2016). Indeed, the national context acts as a point of reference for the way that citizens assess EU integration. The EU integration process is so far from the reality of some citizens, according to a number of scholars, that these citizens formed their opinions about the EU through much closer trusted sources such as the media (Azrout et al. 2012; De Vreese and Boomgaarden 2006; Hooghe 2003) or national politics (De Vries and Edwards 2009).

There are two principal ways to view the relationship between the national and European levels. In the first, scholars argue that citizens evaluate European integration using their countries' institutions as their standard. Because of a lack of knowledge about EU institutions, national aspects assume more importance than European ones (Anderson 1998). Citizens therefore develop attitudes towards the EU using nation-states as a proxy (Kritzinger 2003). In the second, the literature reveals that there is an inverse relationship between national appraisal and support for the EU. Citizens who are disillusioned by the policies of their nation display stronger support for those of the EU and vice versa (Sánchez-Cuenca 2000). This system of standards also works with the quality of national democracy. Citizens who approvingly assess the democracy of their own country show less support for EU integration (Rohrschneider 2002).

Thus utilitarian, identitarian and national determinants each have a different impact on public Euroscepticism. Specific historical, economic or national contexts have lent some of the factors more power. Several scholars, furthermore, have argued that 'attitudes towards multiculturalism' at the individual level and ‘corruption’ at the country level are the strongest Euroscepticism predictors (Ejrnæs and Jensen 2019). Moreover, recent studies revealed considerable cross-country variation as regards the exit from the EU, which implies that motivations for leaving should be assessed on a country-by-country basis and that the anti-globalisation model is subsumed by anti-multiculturalism and anti-elite models (Ejrnæs and Jensen 2021). Covid-19 has various key implications which could affect the attitude towards the EU. Doubt has been voiced by several member states about how the EU institutions operate during an emergency. Among the countries most affected, for example Italy, scholars have observed that people have become more Eurosceptic because of how the virus has been handled as they feel that the EU institutions and other member states abandoned them (Russack and Blockmans 2020).

Some national governments have gained popularity during the Covid-19 crisis by frequently condemning the EU as being inadequate at facing the challenge. In addition, a number of countries attempted to assemble a bloc of solidaristic members against EU coronavirus decisions. This has affected the public's opinion on EU membership, causing important alterations in their relationship with the EU (Bremer et al. 2021). Several governments have backed the idea of future pledge referendums about EU issues, making the Covid-19 era a fascinating way to understand attitudes towards European integration by comparing attitudes before and afterwards.

Historically, national governments have generally committed to referendums on European issues despite not being required to do so. The political strategy behind these national pledges, driven almost fully by national policy linked to domestic issues, has been examined by scholars (Oppermann 2013).

In the past, some EU citizens have had the chance to vote in referendums on European integration. Over 50 referendums have taken place on European issues such as treaty reforms (Mendez et al. 2014). However, the place of EU referendums in the political bond between citizens and institutions can be seen in two different ways. Firstly, scholars have described how EU referendums are understood as second-order ballots on national political issues (Franklin et al. 1995) and secondly, it has been argued that citizens' choices are informed by their own attitude towards the EU (Hobolt 2009).

In this regard, this paper aims to understand Italians' attitude towards the EU, in particular looking at their hypothetical choice of a referendum about the exit from the EU.

In order to understand this, the paper analyses the Eurosceptic component in all member states to then focus on the specific Italian case. From this perspective, this study will analyse the EES survey dataset through the use of categorical principal component analysis (CATPCA). CATPCA has rapidly gained attention among scholars for the purpose of analysing categorical data (i.e. ordinal and nominal data). Similarly, this study applied CATPCA to identify the component structure of the citizens' attitudes by taking into account the ordinal and nominal nature of the data collected in the EES survey. This method allowed us to reduce the data containing significant variables into one or more components that can be used in other analyses.

## Methods and analysis

This paper is based on the 2019 European Election Study (EES) Voter Study conducted in all 28 EU member states after the election to the European Parliament (Schmitt et al. 2020). The post-electoral survey consists of more than 100 items. It was conducted by Gallup International and the data collection was mostly conducted online. The respondents were selected randomly from access panel databases using stratification variables. The sample size was roughly 1000 interviews in each EU member state (except for Cyprus, Luxembourg and Malta where the sample size was 500). The total sample size was 26,538.

The analysis presented in this study considered 21 variables of the EES dataset (Table 1), taking into account five aspects: (a) the national politics focus (Q3, Q5, Q16, Q18_1, Q19 and Q20), (b) EU politics focus (Q4, Q18_2, Q22, Q23 and Q28), (c) the citizens’ attitude towards general policy issues (from Q14_1 to Q14_6), (d) the citizens’ attitudes towards politics (Q8, Q21) and (e) the sociodemographic aspect (HAGE & D11).

**Table 1:** Selected variables

Variable	Label	Type of data
Q3	On the whole, how satisfied are you with the way that democracy works in [country]? Are you…?	Likert scale, 4 points: 1 very satisfied, 2 fairly satisfied, 3 not very satisfied and 4 not at all satisfied
Q4	All in all, again, are you very satisfied, fairly satisfied, not very satisfied or not at all satisfied with the way that democracy works in the European Union?	Likert scale, 4 points: 1 very satisfied, 2 fairly satisfied, 3 not very satisfied and 4 not at all satisfied
Q5	Let us now come back to [country]. Do you approve or disapprove of the government’s record to date?	Binary, 2 categories: 1 approve and 2 disapprove
Q8	How closely did you follow the campaign ahead of the European Parliament elections in the media or on social media?	Likert scale, 11 points: 0 not at all–10 very closely
Q14_1	What do you think of the state regulations and the control of the economy?	Likert scale, 11 points: 0 you are fully in favour of state intervention in the economy–10 you are fully opposed to state intervention in the economy
Q14_2	What do you think of the redistribution of wealth?	Likert scale, 11 points: 0 you are fully in favour of redistribution from the rich to the poor in [country]–10 you are fully opposed to redistribution of wealth from the rich to the poor in [country]
Q14_3	What do you think of same-sex marriage?	Likert scale, 11 points: 0 you are fully in favour of same-sex marriage–10 you are fully opposed to same-sex marriage
Q14_4	What do you think of civil liberties?	Likert scale, 11 points: 0 you fully support privacy rights even if they hinder efforts to combat crime–10 you fully support restricting privacy rights in order to combat crime
Q14_5	What do you think of immigration?	Likert scale, 11 points: 0 fully in favour of a restrictive policy on immigration–10 fully opposed to a restrictive policy on immigration
Q14_6	What do you think of the environment?	Likert scale, 11 points: 0 environmental protection should take priority even at the cost of economic growth–10 economic growth should take priority even at the cost of environmental protection
Q16	How important is it for you to live in a country that is governed democratically?	Likert scale, 11 points: 0 not at all important–10 absolutely important
Q18_1	For each of the following statements, please tell me to what extent it corresponds or not to your attitude or opinion on your trust in the National Parliament	Likert scale, 5 points: 1 yes, totally, 2 yes, somewhat, 3 neither trust nor distrust, 4 no, not really and 5 no, not at all
Q18_2	For each of the following statements, please tell me to what extent it corresponds or does not correspond to your attitude or opinion on your trust in the European Parliament.?	Likert scale, 5 points: 1 yes, totally, 2 yes, somewhat, 3 neither trust nor distrust, 4 no, not really and 5 no, not at all
Q19	What do you think about the economy? Compared to 12 months ago, do you think that the general economic situation in [country] is…?	Likert scale, 5 points: 1 a lot better, 2 a little better, 3 stayed the same, 4 a little worse and 5 a lot worse
Q20	Over the next 12 months, how do you think the general economic situation in [country] will be? Will it…?	Likert scale, 5 points: 1 get a lot better, 2 get a little better, 3 stay the same, 4 get a little worse and 5 get a lot worse
Q21	To what extent would you say that you are interested in politics?	Likert scale, 4 points: 1 very, 2 somewhat, 3 a little and 4 not at all
Q22	Generally speaking, do you think that [country] membership of the European Union is…?	Likert scale, 3 points: 1 a good thing, 2 neither and 3 a bad thing
Q23	Some say that European unification should be pushed further. Others say that it has already gone too far. What is your opinion?	Likert scale, 11 points: 0 unification has already gone too far through to 10 unification should be pushed further
HAGE	Age	Metric, Age (N)
Q28	The British have decided to leave the European Union. What do you think the consequences for the United Kingdom will be, if any?	Multinomial, 3 categories: 1 United Kingdom will be better off, 2 there will be no significant change and 3 United Kingdom will be worse off
D11	Taking everything into account, at what level is your family’s standard of living?	Likert scale, 7 points: 1 poor family–7 rich family

*Source* EES Voter Study 2019 (Schmitt et al. 2020)

The study considered the whole sample of 26,538 starting cases which was subsequently reduced to 16,546. Regarding the Italian case, the sample reduced from 1000 cases to 716.^2^

In summary, this analysis aims to identify the set of indicators for the dimensions relating to the attitudes of Italian citizens in the EES sample towards national and EU issues and politics in general; to test the validity of the structure of the dimensions identified; to hypothesise the different citizens’ profiles through a cluster analysis; and to evaluate the significance of the clusters in relation to both sociographic and political variables before finally testing the impact of the ‘Italexit’ referendum. In particular, the significance of these explanatory factors will be explored in the EU-exit context, such as Brexit, which can be considered a case of ‘exit scepticism’ (De Vries 2018) or ‘hard Euroscepticism’ (Ejrnæs and Jensen 2021).

Upon analysing the dataset, CATPCA is used to identify the components underlying the set of qualitative and ordinal observed variables. The main objectives when choosing CATPCA for the analysis of the data concerned involve (a) a reduction in the data complexity, (b) the exploration of the relationships between the 21 variables observed and (c) the identification of the underlying latent structure.

CATPCA uses optimal scaling procedures to ‘enhance’ the qualitative variables, allowing for the application of the analysis of the main components. For each type of nominal (dummy) and ordinal variable observed (with the order of the modes preserved), the values for the structure have a monotonic function, aiming to maximise the variance between the alternative modes given certain assumptions.

The analysis presents a number of iterations equal to 100, satisfying Cronbach’s Alpha levels (the highest reaches 0.798 with over 19% of variance explained). There are six components to be extracted by applying the Kaiser rule. This was checked using eigenvalues through parallel analysis (Horn 1965) which reports six components and the use of the Scree plot with four components (Table 2).

**Table 2:** Model summary

Dimension	Cronbach’s alpha	Variance accounted for	
		Total (eigenvalue)	% of variance
1	.798	4.168	19.849
2	.582	2.245	10.691
3	.514	1.960	9.336
4	.409	1.638	7.801
5	.156	1.175	5.596
6	.018	1.017	4.843
7	− .119	.898	4.275
Total	.970^a^	13.102	62.391

^a^Total Cronbach’s Alpha is based on the total eigenvalue

*Source* EES Voter Study 2019 (Schmitt et al. 2020)

The outcome of the analysis suggests that the best solution is a five-component one that is to be assessed through the interpretation of the factor coefficients. Subsequently, we applied an oblique rotation method (e.g. Promax) by admitting that there is a possible correlation of the components/factors with each other (due to the similarity of some of the variables). Bartlett’s test is significant (*p* = 0.000) and the KMO index (Kaiser–Meyer–Olkin Measure of Sampling Adequacy) is equal to 0.773. Therefore the factorial model is adequate enough to use to analyse the data. Regarding the communities (which indicate the part of the variance of the individual indicators which remains explained by the factorial model despite the reduction of the ‘*p*’ components and the consequent loss of information), for almost all the indicators, the value is above the critical threshold of 0.50. In fact, the five-component extraction shows that 53.2% of the variance is explained in cumulative terms.

From a first analysis of the pattern coefficient matrix (Table 3), the five-component solution does not show relevant cross-loading. The first component seems to represent the Eurosceptic claims with a strong and negative representation of all variables focused at European level: EU membership is a bad thing, no trust in the EU institutions or how they work in general and a better perspective of the UK after the Brexit referendum. To these variables is added active and full support of a restrictive policy on immigration. The second component seems to be better focused on the national perspective of a pessimistic vision of domestic economic trends related to the past and future. Moreover, this component involves more unfavourable perceptions about the government’s results and no trust in the National Parliament. The third component is more focused on the highest level of interest in politics and the capacity to collect more political information in a country that is governed democratically. The last two components are slightly interpenetrating through some of the cross-loading values. Despite this, the fourth component seems to be closer to a conservativevision in terms of the four critical aspects: being of a higher age, full support of the restriction of privacy rights in order to combat crime, an opposition to same-sex marriage and the belief in the importance of economic growth against environmental protection. The last component is more focused on financial aspects such as the opposition to the state’s intervention in the economy and the redistribution of wealth from the rich to the poor. This component represents the highest level of a family’s standard of living.

**Table 3:** Pattern matrix^a^

	Component				
	1	2	3	4	5
Q22. Generally speaking, do you think that [country] membership of the European Union is…?	.810	.001	− .027	.014	.066
Q18. Do you trust the European Parliament?	.754	.150	.051	.022	− .051
Q23. Some say that European unification should be pushed further. Others say that it already has gone too far. What is your opinion?	− .752	.067	− .024	− .067	.166
Q28. The British have decided to leave the European Union.What do you think the consequences for the United Kingdom will be, if any?	− .746	.123	− .017	.037	− .101
Q4. All in all, again, are you very satisfied, fairly satisfied, not very satisfied or not at all satisfied with the way that democracy works in the European Union?	.638	.223	.088	.049	− .122
Q14. What do you think of immigration?	− .428	.129	− .052	− .096	.354
Q19. What do you think about the economy? Compared to 12 months ago, do you think that the general economic situation in [country] is…?	− .082	.811	.009	.042	.026
Q20. Over the next 12 months, how do you think the general economic situation in [country] will be? Will it…?	− .071	.792	.032	.043	.014
Q5. Let us now come back to [country]. Do you approve or disapprove of the government’s record to date?	− .057	.752	− .006	− .090	.120
Q3. On the whole, how satisfied are you with the way that democracy works in [country]? Are you…?	.135	.710	− .029	− .065	− .050
Q18. Do you trust the … National Parliament?	.168	.675	− .048	− .116	.057
Q21. To what extent would you say you are interested in politics?	− .110	.014	− .869	.102	− .101
Q8. How closely do you follow the campaigns ahead of the European Parliament elections in the media or on social media?	.055	− .020	.851	− .085	.193
Q16. How important is it for you to live in a country that is governed democratically?	− .265	.048	.368	− .072	− .365
Q14. What do you think of civil liberties?	− .071	− .043	− .150	.678	.097
Q14. What do you think of same-sex marriage?	.103	− .088	− .026	.574	.376
Q14. What do you think of the environment?	.178	− .047	− .080	.570	.048
HAGE. Age recode	− .085	.059	.255	.505	− .401
Q14. What do you think of the redistribution of wealth?	− .084	.017	.102	.360	.652
Q14. What do you think of the state regulations and the control of the economy?	− .094	.239	.080	.230	.624
D11. Taking everything into account, at what level is your family’s standard of living?	− .021	− .181	.297	− .118	.470

Extraction methods: Principal component analysis; rotation method: Promax with Kaiser normalisation. ^a^rotation converged in six iterations

*Source* EES Voter Study 2019(Schmitt et al. 2020)

The five components have been renamed in the following way: Euroscepticism (1), National pessimism (2), Knowledgeability (3), Conservatism (4) and Economic liberalism (5).

The factorial scores obtained and standardised were used for cluster analysis. In this case, a k-means cluster analysis with PCA factors as the input was used to identify the profiles of European citizens concerning their attitude towards the aspects analysed before. This type of analysis allows us to maximise the similarity between the elements inside the groups and the dissimilarity between the groups. In other words, the k-average (non-hierarchical) method enables us to maximise the internal and external variance (in and between groups). It is defined a priori as a cluster range (from 2 to 7) taking into consideration the values of the F test, the interpretability of the clusters based on the final centres of the groups (i.e. the averages of the clusters concerning the grouping variables), the number of clusters and their homogeneity and the calculation of each Pseudo-F.

Based on the evaluation criteria, the most reliable solutions were for two, three and four clusters. Pseudo-F has decreasing values (cluster 2 = 3704.57; cluster 3 = 3809.58; cluster 4 = 3523.93). A four-cluster solution was opted for while taking into account its higher homogeneity than the other two. Table 4 shows the four clusters identified.

**Table 4:** Final cluster centres

	Cluster			
	1	2	3	4
Euroscepticism	− .48716	− .58981	− .00603	1.44578
National pessimism	− .04744	− .27357	− .26839	.88969
Knowledgeability	.30279	.64235	− .79955	− .13735
Conservatism	− 1.26206	.73406	− .11376	.25716
Liberalism	− .46009	− .25177	.90357	− .48851
*Number of cases in each cluster*	3249	*5204*	*4855*	*3238*

*Source* EES Voter Study 2019 (Schmitt et al. 2020)

Cluster 1 shows a higher sensitivity towards the ‘Knowledgeability’ component (score 0.30, positive) and little relevance to the ‘Conservatism’ aspect (score 1.26, negative). This cluster is defined as ‘Progressive’.

Cluster 2 shows sensitivity towards the ‘Conservatism’ component (score 0.73, positive) but in this case, there is also the ‘Knowledgeability’ component (score 0.64, positive), although it is less sensitive towards the ‘Euroscepticism’ component (score -0.58, negative). This cluster is defined as ‘Moderate’.

Cluster 3 shows a keen sensitivity towards the ‘Economic liberalism’ component (score 0.90, positive) and a weak relationship with the ‘Knowledgeability’ component (− 0.79). This cluster is defined as ‘Homo economicus’.

Finally, Cluster 4 shows a keen sensitivity towards the ‘Euroscepticism’ component (standardised score of 1.44, positive, the highest value among the four clusters) and also, although as not high as the European level, a negative domestic vision (score 0.88, positive, the highest among the three clusters). It does not seem as interested in the ‘Economic liberalism’ component (− 0.48). This cluster is defined as the ‘Eurosceptic’ cluster.

At this point of the analysis, the data was based only on the Italian case and the four clusters identified were analysed using some of the essential descriptive variables present in the EES questionnaire: sociographic (age, education, area of origin and social class), political (left–right and the probability of voting for a party) and position on the exit referendum.

## The Italian approach to the EU

The identification of the four clusters allows us to analyse the Italian case in more depth in order to understand how the attitudes of Italian citizens are oriented to the dimensions explored in this study. Table 5 presents a bivariate analysis of the four clusters concerning the two types of variables. First, the sociographic group of variables, such as gender, level of education and the urban–rural dimension, represent the social class of belonging. Second, there is the group of political variables such as left–right self-placement, the probability of voting for one of the leading Italian parties and finally the vote for the Italexit referendum.

**Table 5:** The sociopolitical characteristics of the Italian clusters (%)

	Progressive (N = 155)	Moderate (N = 224)	Homo economicus (N = 169)	Eurosceptic (N = 168)	*Total (N* = *716)*
Male	49.7	54.9	52.1	53.6	*52.8*
Female	50.3	45.1	47.9	46.4	*47.2*
15 years and less (= ʻlow’)	2.6	13.4	8.3	10.7	*9.2*
16–19 years (= ʻmedium’)	41.9	42.9	40.2	57.7	*45.5*
20 + (= ʻhigh’)	40.6	41.5	40.2	31.5	*38.7*
still studying	2.6	1.3	2.4	0.0	*1.5*
N/A	12.3	0.9	8.9	0.0	*5.0*
Rural area or village	13.5	15.6	16.0	14.3	*14.9*
Small or middle-sized town	50.3	56.3	47.3	54.8	*52.5*
Large town	36.1	28.1	36.7	31.0	*32.5*
Working class	7.1	7.1	10.1	14.9	*9.6*
Lower middle class	27.1	20.1	18.9	32.7	*24.3*
Middle class	51.0	60.7	56.8	44.0	*53.8*
Upper middle class	10.3	11.6	9.5	7.7	*9.9*
Upper class	1.9	0.4	3.0	0.0	*1.3*
Other	2.6	0.0	1.8	0.6	*1.1*
Left	29.7	11.6	2.4	4.8	*11.7*
Centre-left	33.5	22.3	16.0	11.3	*20.7*
Centre	11.6	17.9	20.1	16.7	*16.8*
Centre-right	15.5	30.8	44.4	24.4	*29.2*
Right	6.5	14.7	11.2	34.5	*16.8*
Don’t know	3.2	2.7	5.9	8.3	*4.9*

As for the first group of variables, there is no significant difference in relation to the gender variable except for the ‘Progressive’ cluster which has a slight female majority (50.3%). It has also been seen (see Tables 3 and 4) how age is used as a metric variable within the model that has characterised the ‘Conservatism’ component positively and the ‘Economic liberalism’ component negatively. Consequently, the relative clusters built on the five components are identified by age; the group ‘Moderate’ involves older people and the ‘Homo economicus’ group involves younger people. Furthermore, age influences the other two groups, albeit to a lesser extent. The ‘Progressive’ group is composed of a group of younger citizens while the ‘Eurosceptic’ group is populated by older people.

In terms of education, two critical aspects have emerged from the data. The first concerns the ‘Progressive’ group that is mainly anchored to the ‘Knowledgeability’ factorial component which has the lowest level of ‘low education’ (2.6%) and consequently the highest percentage in the medium–high levels. The second concerns the ‘Eurosceptic’ cluster which presents the lowest percentage of ‘highly’ schooled citizens (31.5%). So far as professions are concerned, the highest rate of people in the working class (14.9%) and lower middle class (32.7%) are recorded in the ‘Eurosceptic’ group. At the same time, the ‘Progressive’, ‘Moderate’ and ‘Homo economicus’ clusters present with population groups belonging to the medium–upper classes. In the urban–rural dimension, all clusters show other interesting details. The ‘Progressive’ and ‘Homo economicus’ groups mainly belong to middle- to large-sized towns while the ‘Moderate’ and ‘Eurosceptic’ groups belong to middle- to small-sized towns or rural areas.

Regarding the political variables, there is a higher placement of citizens close to the left and centre-left areas in the ‘Progressive’ cluster, gradually passing through the ‘Moderate’ and ‘Homo economicus’ groups that are more representative of the central areas of the left–right self-placement in order to get to the ‘Eurosceptic’ group in a centre-right or extreme right position. Consequently, the propensity to vote for one of the seven parties in the EES survey also varies according to the cluster and ideological self-placement (Table 6). For this reason, the table shows that for each political party, the data relating to the average values have been recorded by each cluster on a scale ranging from 0 to 10 where 0 indicates no probability of voting for that party and 10 certain probability. In this regard, the ‘Progressive’ group has a high likelihood of supporting the centre-left parties (i.e. 7.2 for PD or 4.8 for the Left party ‘Sinistra’). The ‘Moderate’ group seems to be very supportive of the positions of both the PD (8.8) and the League (8.5) while the ‘Homo economicus’ group are ‘Eurosceptic’ and firmly centred on the League (i.e. 8.2 and 9.2). In short, Salvini’s League registers in three of the four groups a higher probability of gaining the vote.

**Table 6:** Propensity to vote of the Italian clusters (0–10 scale)

	Progressive (N = 155)	Moderate (N = 224)	Homo economicus (N = 169)	Eurosceptic (N = 168)	*Total (N* = *716)*
PD	7.2	8.8	6.3	1.5	*5.9*
FI	2.3	5.7	6.1	3.7	*4.5*
League	2.6	8.5	8.2	9.2	*7.2*
M5S	4.6	6.9	7.7	6.6	*6.5*
Sinistra	4.8	3.9	4.5	1.0	*3.6*
+ Europa	5.6	5.8	5.7	1.2	*4.6*
FdI	2.0	6.6	6.2	5.7	*5.1*

The last variable indicates the position of the clusters in the face of a hypothetical referendum on exiting the EU (Table 7). Overall, the Italian sample highlights a clear majority for the ‘remain’ position with a higher percentage in the ‘Progressive’ and ‘Moderate’ groups at 83.9 and 86.6% respectively. However, two aspects seem relevant to better understanding the hypothetical referendum. First, despite the fact that the majority in the ‘Homo economicus’ group are focused on the ‘remain’ position (55.6%), it is important to show how the group represents the highest percentage of people who are likely to vote for neither of the two views (26%). This is possible due to having both inadequate information about the EU and how it works and self-interest based mainly on economic convenience. These factors influence the decision to stay in the EU until the benefits outweigh the costs. The risk is that a reversed trend may follow a change in this as yet uncrystallised position. Second, there is an evident prevalence of the ‘leave’ position in the ‘Eurosceptic’ group (70.8%). The data indeed allows for exploring the ‘hard/exit scepticism’ explained by De Vries (2018) and Ejrnæs and Jensen (2021). In brief, in a group characterised mainly by the ‘hard/exit scepticism’ factor, the choice to remain within the EU (18.5%) seems to overlap with an idea of a softer Euroscepticism.

**Table 7:** The position on the Italexit referendum of the Italian clusters (%)

	Progressive (N = 155)	Moderate (N = 224)	Homo economicus (N = 169)	Eurosceptic (N = 168)	*Total (N* = *716)*
Remain a member of the EU	83.9	86.6	55.6	18.5	*62.7*
Leave the EU	10.3	9.4	18.3	70.8	*26.1*
Neither remain nor leave, of which:	5.8	4.0	26.0	10.7	*11.2*
You would submit a blank ballot paper	2.6	1.3	11.8	0.6	*3.9*
You would spoil the ballot paper	1.9	0.0	5.3	1.2	*2.0*
You would not vote	0.6	0.0	2.4	3.6	*1.5*
You are not eligible to vote	0.6	0.0	0.0	0.6	*0.3*
Don’t know	0.0	2.7	6.5	4.8	*3.5*

The other groups also present as having a significant percentage of citizens willing to leave the EU. In this case, it is not the Eurosceptic component that guides their ideas but either the ideological dimension or the economic one.

To summarise, the ‘Progressive’ group is characterised by a more significant female presence and high levels of education, well-heeled and residing in large urbanised centres. This cluster tends to vote for centre-left parties with a higher percentage of the vote supportive of remaining a member of the EU. The moderate cluster is concentrated in medium urbanised areas and it is composed of the middle class with an average income. It is self-placed in mainly centrist ideological positions and does not disdain cross-voting for both the PD and the League despite having the highest percentage of people who are pro-EU membership. The ‘Homo economicus’ cluster is divided between the rural area and the large-sized town; it is mainly centre-right and it is close to the League and M5S positions. It is generally in favour of staying in the EU but it also has the highest percentage of people who are not likely to take part in the event of a referendum.

Finally, there is the ‘Eurosceptic’ cluster with the lowest levels of education and the most moderate incomes who are residents in the peripheral areas of the country far from centre-left political views and, above all, oriented to the League’s position. It is possible to analyse the clear negative position against Europe. However, a percentage of people in this cluster are likely to vote to remain in the EU.

## Conclusion

This study aims to understand how Euroscepticism takes root in Italian citizens and how they are likely to react in the face of a hypothetical referendum on leaving the EU based on the model of the one held in the UK.

While the UK is considered an outlier regarding the exit from the EU (Hobolt 2016), recent studies have highlighted how there are different paths to the exit from the EU among countries and that anti-multiculturalism and anti-establishment factors have an explanatory power to account for Euroscepticism in Italy (Ejrnæs and Jensen 2021).

This implies that assumptions about a hypothetical Italexit must be considered sensitive to the heterogeneity of these results instead of assuming that the same set of causes could influence all other member states equally, although this kind of analysis could be tested in future for all others.

The results, indeed, highlight that the ‘Euroscepticism’ component contains a piece of clear evidence in relation to the sample analysed. Often it is associated with a negative vision of the national politics and it is opposed to the ‘Knowledgeability’ and ‘Conservatism’ components. It seems not to display sensitivity to the ‘Economic liberalism’ component (see Table 4). Consequently, the ‘Euroscepticism’ component appears to define a whole cluster of Eurosceptic people with a hard (or softer) scepticism approach, although this cluster is smaller than the others and also has national aspects. Moreover, observing the UK experience allows Italians to better evaluate the feasibility and advantage of an EU withdrawal. Recent studies show how more positive withdrawal experiences encourage exit-support abroad, whereas negative experiences are likely to deter (Walter 2020). These contagion effects impact EU membership and its long‐term stability, increasing the Eurosceptic view if the perception of Brexit negotiations is as being positive for the UK, allowing the development of a blueprint that makes it easier for countries to leave the EU in the future (Walter 2021).

The specific Italian case has a ‘Eurosceptic’ cluster defined by low levels of education, a rural area of origin, a lower middle social class and being close to the centre-right position, in particular the League. This profile shows that the M5S has lost attractiveness as a Eurosceptic political reference. But why? One important reason was that Salvini’s League prevailed in two crucial issues, one of which is very relevant and essential to the Eurosceptic component. The first is the synergy between immigration and security which he trusts more than other Italian politicians. Second, Salvini has always been perceived as the protector of Italian interests and as a proponent of ‘Italy first’. This is a component that is very relevant in the ‘national pessimism’ analysed in this study. Moreover, Salvini is an experienced politician who has dominated the government and now dominates the opposition. His attention is mainly focused on the possibility of gaining government positions again. At the moment, the League of Salvini is influential in Italy.

In this context, the tension between the government and opposition has focused on the EU issue. The League, M5S and also the FdI have tried to characterise themselves in the EU as the defenders of the Italian people, threatened from above by the elites and from below by many dangerous ‘others’, especially immigrants. The parties are trying to show that they have an international political role. Each political party represents an alternative to the other, thinking that their time has come and believing that they are part of a wave that will sweep the world and change both politics and its actors.

In this state of political competition, the Italexit referendum still seems to be a distant danger. The majority appears to be oriented towards a ‘remain’ vote with two significant clusters, ‘Progressive’ and ‘Moderate’, almost entirely in favour of this. Something is missing at the ‘Economic liberalism’ cluster level where it is no coincidence that M5S reaches the highest level of representation. Somehow it seems that the confusion within the party that has manifested in recent years concerning the relationship with the EU also seems to have had repercussions on a slice of the population that has looked at the movement carefully. Finally, the ‘Eurosceptic’ cluster is almost entirely driven by ‘leavers’, demonstrating that this position has settled in a part of the population and that it will be essential to monitor in future studies.

New forms of anti-politics and populism, sometimes related to each other and at other times not, with a high level of distrust in politics or the EU and its manifestations, have taken on a karstic trend. This involves running underground and emerging periodically with explicit reference to particularly deplorable events and focusing on the behaviours of the parties, the political class or EU institutions, leaving space for the affirmation of attitudes of despair or anger that find root in a possible referendum on leaving the EU as the (vain) solution to all problems.

## Footnotes

1 Several parties have discussed it in their electoral manifestos, such as the M5S with the promise of a referendum on the permanence of the euro in 2014 or the League in its 2018 and 2019 manifestos.

2 To obtain an optimal CATPCA, the sample was reduced for the following reasons: (1) the items related to the questions presented an interval scale of several points from 2 to 11 beyond 'don't know' and missing values; (2) it eliminated the cases where the 'don't know' mode and the missing values were present as they constitute a problem in the statistical approach adopted (variables that could not be ordered); (3) the choice led to 'purifying' the dataset of total cases compared to the original, making the variables 'ordinal'; (4) for the remaining 16,546, a CATPCA was applied with optimal scaling to transform the ordinal variables into metrics; (5) a cluster analysis was conducted on the factors identified and (6) the bivariate analysis used sociopolitical variables to examine the issue of the possible referendum on the exit from the EU.
